# The Role of the Superior Cervical Sympathetic Ganglion in Ischemia Reperfusion-Induced Acute Kidney Injury in Rats

**DOI:** 10.3389/fmed.2022.792000

**Published:** 2022-04-21

**Authors:** Wencui Zhang, Zhen Li, Zhixiao Li, Tianning Sun, Zhigang He, Anne Manyande, Weiguo Xu, Hongbing Xiang

**Affiliations:** ^1^Department of Anesthesiology, Tongji Medical College, Tongji Hospital, Huazhong University of Science and Technology, Wuhan, China; ^2^School of Human and Social Sciences, University of West London, London, United Kingdom; ^3^Department of Orthopedics, Tongji Medical College, Tongji Hospital, Huazhong University of Science and Technology, Wuhan, China

**Keywords:** cervical sympathetic ganglion, superior cervical ganglionectomy, sympathetic nervous system, immune system, renal ischemic reperfusion injury, acute kidney injury

## Abstract

Acute kidney injury (AKI) has been found to be a serious clinical problem with high morbidity and mortality, and is associated with acute inflammatory response and sympathetic activation that subsequently play an important role in the development of AKI. It is well known that the sympathetic nervous system (SNS) and immune system intensely interact and mutually control each other in order to maintain homeostasis in response to stress or injury. Evidence has shown that the superior cervical sympathetic ganglion (SCG) participates in the bidirectional network between the immune and the SNS, and that the superior cervical ganglionectomy has protective effect on myocardial infarction, however, the role of the SCG in the setting of renal ischemic reperfusion injury has not been studied. Here, we sought to determine whether or not the SCG modulates renal ischemic reperfusion (IR) injury in rats. Our results showed that bilateral superior cervical ganglionectomy (SCGx) 14 days before IR injury markedly reduced the norepinephrine (NE) in plasma, and down-regulated the increased expression of tyrosine hydroxylase (TH) in the kidney and hypothalamus. Sympathetic denervation by SCGx in the AKI group increased the level of blood urea nitrogen (BUN) and kidney injury molecule-1 (KIM-1), and exacerbated renal pathological damage. Sympathetic denervation by SCGx in the AKI group enhanced the expression of pro-inflammatory cytokines in plasma, kidney and hypothalamus, and increased levels of Bax in denervated rats with IR injury. In addition, the levels of purinergic receptors, P2X3R and P2X7R, in the spinal cord were up-regulated in the denervated rats of the IR group. In conclusion, these results demonstrate that the sympathetic denervation by SCGx aggravated IR-induced AKI in rats via enhancing the inflammatory response, thus, the activated purinergic signaling in the spinal cord might be the potential mechanism in the aggravated renal injury.

## Introduction

Acute kidney injury (AKI), is a frequent clinical syndrome primarily caused by renal ischemic-reperfusion (IR) injury in situations such as during major surgery, septic shock, cardiogenic shock, hypovolemia, and nephrotoxic drugs (1). AKI has a high risk of morbidity and mortality, as well as progression to chronic kidney disease (CKD) and end-stage renal disease (ESRD), leading to increased resource utilization and social burden (2, 3). The mechanism underlying IR-induced AKI is not fully understood, however, it has been reported that inflammation, apoptosis, oxidative stress and other factors contribute to the pathogenesis of this renal injury (1, 4). A wide array of studies have demonstrated that the kidney is innervated by both afferent sensory nerve fibers and efferent sympathetic nerve fibers, which are activated in renal IR injury (5, 6). It is also well established that a kidney-central nervous system crosstalk exists in the acute setting that renal sympathetic nerve activity arises from and provides context to the central integration with incoming sensory information from the somatosensory and viscerosensory systems that regulate fluid volume and blood pressure (5, 7). A “reno-renal” reflex is formed in which tissue damage associated with ischemia results in activation of primary afferent nerve fiber terminals in addition to the release of the mediators of calcitonin gene-related peptide (CGRP), substance P (SP) and other neuropeptides from the afferent nerve fiber terminals. These transfer signals to the central nervous system followed by improved neural activity of the hypothalamus, a central site for the integration of sympathetic activity, which results in increased sympathetic outflow to the kidney (4, 8). And the renal sympathetic nervous system and circuiting catecholamines are considered to be involved in the development of ischemic acute kidney injury (9).

Over the last few decades, a body of data has continually emerged implicating an inextricable link between the nervous and immune systems. Evidence has demonstrated that sympathetic overactivity aggravates IR-induced renal damage via pro-inflammation mechanisms (10, 11). Activation of the pro-inflammation response is characterized by the so called “cytokine storm,” which is represented by excessive cytokines including tumor necrosis factor-alpha (TNF-α), interleukin (IL)-1β and IL-6 (12). In turn, in the early course of AKI, the various pro-inflammatory cytokines released into the arterial inflow and venous outflow of the kidney (13) can pass through the brain-blood barrier in order to stimulate the paraventricular nucleus (PVN), the rostral ventrolateral medulla (RVLM) and the solitary tract (NTS), which augment sympathetic outflow to the peripheral (14–16). Anti-inflammation actions of the sympathetic nervous system (SNS) also have been demonstrated in several systems (17, 18). The interaction between the SNS and the immune system in AKI-induced by renal IR injury is unclear and is a subject worth in-depth study.

The superior cervical ganglion (SCG) is one of the important parts of the sympathetic nervous system with the most traffic branches and special distribution positions. Bilateral sympathectomy of SCG has been shown to lead to degeneration of the tyrosine hydroxylase (TH) nerve fiber, the rate-limiting enzyme in the biosynthesis of catecholamine and a marker of SNS (19), in dura (20), and major cerebral arteries (21), and the regulation of cerebral blood flow (CBF) (22, 23). Likewise, SCG partly provides noradrenergic innervation of the hypothalamus (24, 25). Removal of the superior cervical ganglion significantly decreased NE uptake in the medial basal hypothalamus (MBH) and the result could possibly be explained by the peripheral sympathetic neurons or fibers from SCG projection to the MBH and/or the changed neuroendocrine activity brought by SCGx (26). Previous studies have demonstrated that after SCGx, there were changes in various hormones synthesized and/or released at hypothalamic nuclei (27–29), some of which have the ability to regulate cardiovascular function such as vasopressin. In fact, suppression of the cervical sympathetic innervation could affect not only the median cerebral structure and hypothalamic-pituitary axis, but also generally alter numerous neuroendocrine system (22, 30).

An earlier study demonstrated that the cervical sympathetic trunk as a relay of the bidirectional communication network between the nervous and immune system, and bilateral ganglionectomy of SCG, notably reduced the pulmonary inflammation potentially mediated by tissues or organs innervated by SCG (31). However, there exist some ambiguity concerning the effect, with a recent study revealed that lipopolysaccharide-induced inflammation was increased after sympathetic denervation (32). Sympathetic hypofunction might lead to an increase in parasympathetic activation and vice versa. The influence of parasympathetic nervous system on immune response has been sought in the light of studies showing that vagus nerve stimulation attenuated inflammation via activating the sensory efferent vagus nerve that suppresses monocyte and/or macrophage production of pro-inflammation cytokines such as TNF-α and IL-6 (33–35). The latest research shows that superior cervical ganglionectomy attenuated myocardial inflammation and cardiac disfunction after myocardial infarction (36). Hence, the role of SCG in the immune system is ambiguous and controversial, and needs further investigation.

In the light of the above research findings, the significance of the superior cervical ganglion in regulating acute renal injury is vague. The sympathetic neuron derived from SCG could modulate the stress-induced hypothalamus neurotransmitter, norepinephrine (NE), which plays a central role in the neuroendocrine response to stress. Thus, we hypothesized whether the superior cervical ganglion plays a role in AKI induced by renal IR injury through adjusting the sympathetic outflow from the hypothalamus and regulating the immune response. Our objective was to test the hypothesis that the “reno-brain axis” interacts via changes in renal afferent and efferent sympathetic nerve activity that contribute to the renal and brain inflammation and to the progression of ischemic AKI.

## Materials and Methods

### Animals

Male Sprague Dawley (SD) rats (180–200 g; specific pathogen-free grade) were purchased from the Laboratory Animal Center of Tongji Medical College, Huazhong University of Science and Technology (Wuhan, Hubei, China). Animals were individually housed in a climate controlled room (temperate of 25 ± 1^°^C, relative humidity of 50 ± 10%, and a 12 h light/dark cycle) with *ad libitum* food and water. All experiments were strictly carried out in accordance with the National Institutes of Health Guide for the Care and Use of Laboratory Animals. The present experimental protocol was approved by the Institutional Ethical Committee of Tongji Hospital, Tongji Medical College, Huazhong University of Science and Technology (Wuhan, China) (TJH-202102002).

### Experimental Design

After 1 week of acclimation, all rats were randomly divided into the following four groups:(1) Sham group (sham, *n* = 6); (2) IR group (IR, *n* = 6); (3) superior cervical ganglionectomy + sham group (SCGx, *n* = 7); (4) IR + superior cervical ganglionectomy group (IR + SCGx, *n* = 7). All surgical procedures were conducted with sterile instruments. For all surgeries, rats were anaesthetized with 40 mg/kg sodium pentobarbital intraperitoneal injection and placed on a heating pad to maintain body temperature. The SCGx surgery was performed as previously described (37). After induction of anesthesia and disinfection, a 2 cm vertical incision was performed at the neck region and then salivary glands were exposed and carefully dissected to expose the underlying muscles. With blunt forceps, the cranial portion of the sternomastoid muscle (SMM) and the omohyoid muscle (OMH) were transected for clear visibility of the common carotid artery (CCA). Next the CCA was bluntly dissected cranially to locate the carotid bifurcation into the external and internal carotid arteries (ECA and ICA), the SCG was identified behind the carotid bifurcation. Finally, the cell body of the ganglion was gently pulled until its full avulsion from the sympathetic chain and the SCG tissue were collected. Complete superior cervical ganglionectomy was achieved by the additional removal of the SCG on the contralateral side. After SCG removal, the incision was closed with 4–0 sutures and compound lidocaine cream was applied to the wound locally to alleviate incision pain. In the sham group, only exposure of the superior cervical ganglion was performed. After surgery, rats were placed back on heated pads for recovery and then returned to their own cages with free access to food and water. The palpebral ptosis was used as an indicator of the successful removal of the SCG. Fourteen days after the intervention, acute kidney ischemic reperfusion (IR) injury was performed according to previous published protocol (38). A midline laparotomy was made to expose the bilateral renal pedicles and the renal IR were induced by clamping both renal pedicles for 45 min with a non-traumatic vascular clamp. The ischemic was confirmed by visual inspection of the kidney from bright red to purple-black. To reduce abdominal air, 1 ml warm normal saline was given intraperitoneally before abdominal closure. In the sham operation group, a similar surgical procedure was performed, except for renal pedicle clamping. All rats were euthanized 24 h after reperfusion, and blood and tissue samples (kidney, T8-T12 spinal cord and hypothalamus) were collected for experimental studies.

### Analysis of Renal Function and Histology

Renal function was detected by measuring serum creatinine (Scr) and blood urea nitrogen (BUN) in plasma at the Department of Clinical Laboratories of Tongji Hospital. For histology, excised left kidney were processed for light microscopic observation, according to the standard procedure. The kidney specimens were first fixed with 10% formalin solution and embedded with paraffin. Then, 3 um thick renal tissue sections were cut and stained with hematoxylin and eosin. Histopathological changes were analyzed and graded as follows: (1) tubular epithelial smoothness or tubular expansion: score 1, (2) loss of brush-like edge: score 1or 2, (3) obstruction of tubular lumen: score 1 or 2, (4) cytoplasmic vacuolization: score1, and (5) cell necrosis: score 1. The evaluation of histological data was performed by two independent observers blinded to the experimental groups.

### Western Blot

Protein from kidney samples was extracted by RIPA lysis containing freshly added phosphatase and protease inhibitors. Protein concentrations were determined using the BCA Protein Assay Kit (Wuhan Boster Biological Technology, Ltd., China). Equal amounts of total protein (30–50 μg/lane) were separated on 10% SDS-PAGE gels and subsequently transferred to PVDF membranes. The membranes were blocked in 5% skim milk in TBST for 2 h at room temperature and then incubated with primary antibody overnight at 4^°^C. Primary antibodies were applied as follows: rabbit anti-TNF-α (1:1000; Cat No. 17590-1-AP; Proteintech Group Co., Ltd., China), rabbit anti-IL-6 (1:1000; Cat No. A0286; Abclonal Technology Co., Ltd., China), rabbit anti-TH (1:1,000; #58844S; Cell Signaling Technology, United States), rabbit anti-Bcl2 (1:1,000; Cat No. A11313; Abclonal Technology Co., Ltd., China), rabbit anti-BAX (1:1,000; Cat No. A0207; Abclonal Technology Co., Ltd., China), rabbit anti-Caspase-3 (1:1,000; Cat No. A19664; Abclonal Technology Co., Ltd., China), rabbit anti-P2X3R (1:1,000; Cat No. A12965; Abclonal Technology Co., Ltd., China), rabbit anti-P2X7R (1:1,000; Cat No. A10511; Abclonal Technology Co., Ltd., China), rabbit anti-GAPDH (1:10,000; Cat No. BM1623; Wuhan Boster Biological Technology, Ltd., China), rabbit anti-β-actin (1:100,000; Cat No. AC026; Abclonal Technology Co., Ltd., China). After washing, the membrane was incubated with HRP-conjugated goat-anti-rabbit IgG (1:5,000; Cat No. BA1065; Wuhan Boster Biological Technology, Ltd., China) for 2 h at room temperature, bands were visualized using Super-Lumia ECL Plus HRP Substrate Kit (K22030; Abbkine Scientific Co., Ltd., China) and detected using a computerized image analysis system (ChemiDoc XRS1, Bio-Rad, Hercules, CA). The mean intensities of selected areas were normalized to values of the internal control (GAPGH and β-actin) and the areas of these images were calculated using the image lab software (Bio-Rad).

### Immunofluorescence Staining

After removal, the SCG was fixed with PBS in 4% paraformaldehyde overnight at 4^°^C. The ganglia were washed three times in PBS for 5 min per wash, dehydrated in 20% sucrose/PBS for 24 h and 30% sucrose/PBS for 48 h and embedded in OCT. 20 μm thick serial sections of the ganglia were cut with a Leica cryostat. These sections underwent three 10-min washes in PBS and permeabilized with PBST (0.3% TritonX-100 in PBS) for 30 min. After washing, the tissue slices were incubated with blocking solution (5% bovine serum albumin) for 1 h at room temperature. Then sections were incubated at 4^°^C overnight with the rabbit anti-TH antibody (1:100; #58844S; Cell Signaling Technology, United States). After three washes in PBS for 10 min per wash, sections were incubated with secondary antibody conjugated with Alexa Fluor 488 (Goat Anti-Rabbit IgG, 1:100; Protomer) for 2 h at room temperature followed by counterstaining with DAPI (Wuhan Boster Biological Technology, Ltd., China). The sections were examined using fluorescence microscope (DM2500; Lecia).

### Enzyme-Linked Immunosorbent Assay

Blood samples were collected (1.5 ml) from the postcava into heparinized tube and then centrifuged at 3,000 rpm for 10 min at 4^°^C. The supernatants were collected, and the levels of plasma IL-6, TNF-α, NE were determined using the Rat IL-6R ELISA kit (Cat No. E-EL-R0896c; Elabscience Biotechnology Co., Ltd., China), Rat TNF-α ELISA kit (Cat No. E-EL-R2856c; Elabscience Biotechnology Co., Ltd., China) and Noradrenaline/Norepinephrine ELISA kit (Cat No. E-EL-0047c; Elabscience Biotechnology Co., Ltd., China). The procedures were according to the manufacturer’s instructions, and the concentration was presented as pg/ml.

### Real-Time PCR

Total RNA from the kidney, hypothalamus or spinal cord (T8-T12) was extracted by Trizol (Takara, Japan), and then the RNA concentration was quantified by a spectrophotometer (Eppendorf, Germany). RNA reverse transcription was conducted using PrimerScript™ reagent kit (Takara, Japan) according to the manufacture’s instruction. TNF-α, IL-6, NGAL, Kim-1, TH, P2X3R, P2X7R were amplified using ChamQ Universal SYBR qPCR Master Mix (Vazyme Biotechnology Co., Ltd., China) on the ABI7900 (Illumina). The relative expression level of mRNA was quantified by normalization to β-actin of the Sham group and analyzed with 2^–ΔΔCT^ method. The sequences of the primers are listed in Table 1.

**TABLE 1:** Primers used for real-time PCR.

Primer name	Forward primer (5′→3′)	Reverse primer (3′→5′)
TNF-α	AAAGGACACCATGAGCACGGAAAG	CGCCACGAGCAGGAATGAGAAG
IL-6	ACTTCCAGCCAGTTGCCTTCTTG	TGGTCTGTTGTGGGTGGTATCCTC
KIM-1	ATAGTGGTCTGTATTGTTGCCGAGTG	TGTGGTTGTGGGTCTTGTAGTTGTG
NGAL	TTGACAACTGAACAGACGGTGAGC	GAAAGATGGAGCGGCAGACAGAC
TH	TTGACCCTGATCTGGACCTGGAC	ATTGGTTCACCGTGCTTGTACTGG
P2X3R	AAAGAGATGTGGGAGAGGGAGAGTC	GTGGGCAAGCAGAGGGAAGAAAG
P2X7R	ATTAACCAGACTAGAAGCCATCGCATC	AGCAGTCACTTAGAACCATAGCATAGC

*TNF-α, tumor necrosis factor-alpha; IL-6, interleukin-6; KIM-1, kidney injury molecule-1; NGAL, neutrophil gelatinase-associated lipocalin; TH, tyrosine hydroxylase; P2X3R, purinergic receptors P2X3; P2X7R, purinergic receptors P2X7.*

### Statistical Analyses

All data are present as mean ± SEM, *P-*value < 0.05 was considered statistically significant. We used one-way ANOVA followed by the Bonferroni test for statistical analysis with the GraphPad Prism 8.0 software.

## Results

### Sympathetic Denervation by Superior Cervical Ganglionectomy Effectively Reduced Levels of Norepinephrine in Plasma and the Expression of Tyrosine Hydroxylase in the Kidney and Hypothalamus After Ischemic Reperfusion Injury

**FIGURE 1:**
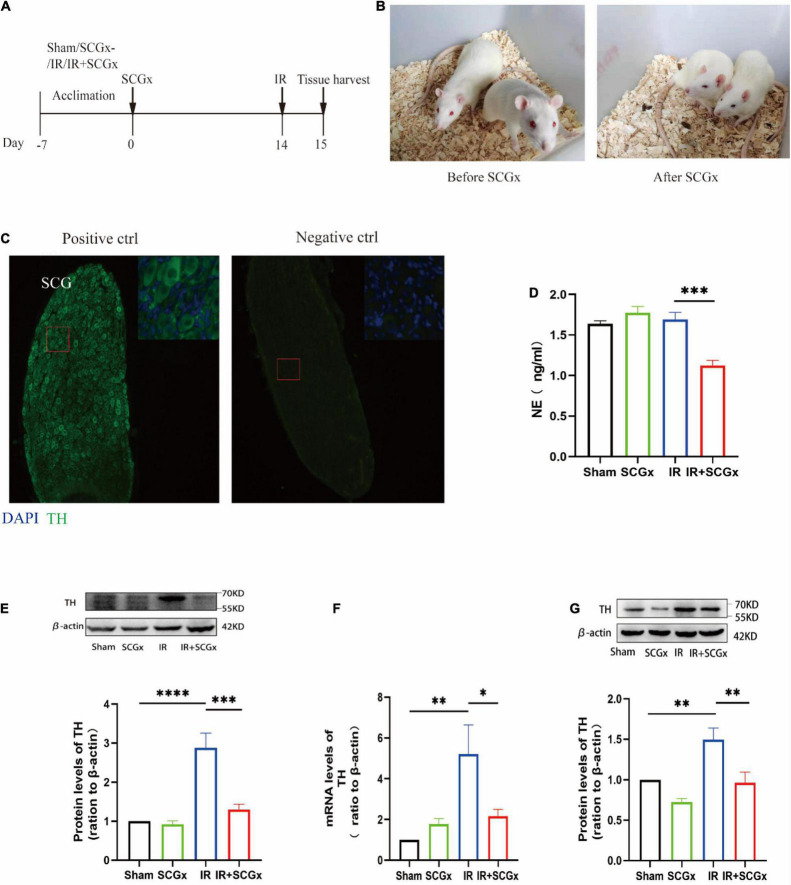
Surgical removal of SCGs in IR rats reduced sympathetic activity. **(A)** Experimental scheme. The intervention group included Sham, SCGx (bilateral removal of the SCG), IR (bilateral renal ischemic for 45 min and reperfusion for 24 h), and IR + SCGx (bilateral removal of the SCG followed by renal ischemic reperfusion surgery). Tissue harvesting was performed after 24 h reperfusion. **(B)** Bilateral blepharoptosis after bilateral superior cervical ganglionectomy. **(C)** Immunofluorescence staining of surgically removed SCG. The same staining procedure served as a negative control, except that the primary antibody was omitted. Tyrosine hydroxylase (TH). Scale bar: 50 μm. **(D)** The NE concentration in plasma by ELISA. **(E)** The protein levels of TH in the kidney. β-actin served as an internal control. **(F)** The mRNA levels of TH in the hypothalamus. β-actin served as an internal control. **(G)** The protein levels of TH in the hypothalamus. β-actin served as an internal control. *P < 0.05; **P < 0.01; ***P < 0.001; ****P < 0.0001, each group contains at least 4 rats.

The experimental outline is depicted in Figure 1A. Fourteen days prior to renal IR surgery, superior cervical ganglionectomy (SCGx) was conducted, and the animals were randomly assigned into the following four groups, i.e., a sham group (sham), a sympathetic denervation group (SCGx), a renal ischemic reperfusion group (IR) and a renal ischemic reperfusion group with sympathetic denervation group (IR + SCGx). Palpebral ptosis is usually used as an indicator to assess the effectiveness of the SCGx and the rat with bilateral blepharoptosis was apparent after surgery (Figure 1B). In addition, we stained cryosection with antibodies against TH, a marker for SNS neurons (19), in order to validate that the removed tissue indeed contained the ganglionic sympathetic neurons (*n* = 6). All of the removed structure stained positive for TH with no signal detectable in the absence of the primary antibody (Figure 1C). We then sought to determine the effect of sympathetic denervation on the SNS and examine the expression of TH in the kidney and hypothalamus. As shown in Figure 1D, marked reduction of NE concentration was observed in the IR + SCGx group in plasma, compared with IR rats (1.235 ± 0.08399 ng/ml vs. 1.641 ± 0.08838 ng/ml, *P*<0.001). The protein levels of TH were significantly increased in the kidney after IR injury, as compared with the sham control (Figure 1E). However, sympathetic denervation markedly decreased TH levels after IR injury (2.877 ± 0.3784 vs. 1.3 ± 0.1356, *P <* 0.0001, Figure 1E). Consistent with the results in the kidney, the mRNA and protein levels of TH in the hypothalamus were also determined and remarkedly increased in renal IR animals, compared with the sham-operated rats (Figures 1F,G). However, levels were significantly reduced in the denervated rats with renal IR-induced AKI (mRNA 2.155 ± 0.3414 vs. 5.204 ± 1.434, *P <* 0.05, Figure 1F; protein 0.9622 ± 0.1333 vs. 1.495 ± 0.1441, *P <* 0.05, Figure 1G). Taken together, the results above demonstrate that sympathetic denervation by SCGx resulted in a drastic reduction in NE content of plasma and reduced expression of TH in the kidney and hypothalamus after IR injury.

### Sympathetic Denervation Contributed to Renal Functional and Structural Impairment After Ischemic Reperfusion Injury

**FIGURE 2:**
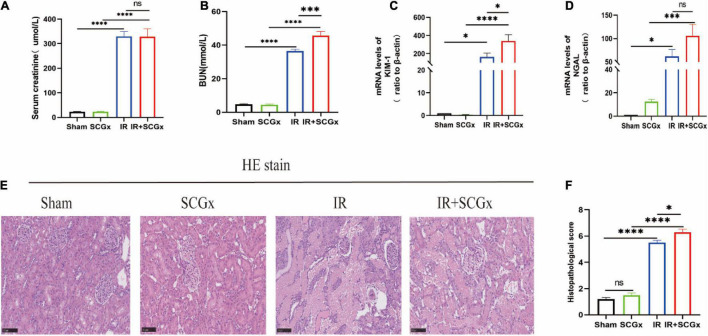
Sympathetic denervation aggravated renal IR injury. **(A)** The Scr levels. **(B)** The BUN levels. **(C,D)** The mRNA levels of KIM-1 and NGAL in kidney. β-actin used as internal control. **(E)** Representative images of H&E staining in rat kidneys. **(F)** Histological score from H&E samples (*n* = 10). Scale bar = 100 μm. *P < 0.05; ***P< 0.001; ****P < 0.0001, each group contains at least 4 rats.

Compared with sham-operated rats, the renal function of rats allocated to the IR group showed a marked deterioration with significant increase in Scr and BUN concentration (Figures 2A,B). In addition, the increased BUN induced by renal IR injury was significantly enhanced by sympathetic denervation in ischemic acute kidney injury rats (36.49 ± 1.031 mmlo/l vs. 45.74 ± 2.398 mmol/l, *P* < 0.001, Figure 2B). However, there was no difference in Scr concentration between the IR group and IR + SCGx group (330 ± 19.14 μmol/L vs. 328.3 ± 32.12 μmol/L, *P* > 0.05, Figure 2A). To confirm the renal injury further, we also detected the Neutrophil Gelatinase-Associated Lipocalin (NGAL) and Kidney Injury Molecule-1 (KIM-1) in the kidney and found a notable enhancement in rats from the IR group, compared with sham-operated rats. Rats with AKI induced by IR injury treated with sympathetic denervation had higher levels of KIM-1 (339.2 ± 69.8 vs. 165.2 ± 40.98, *P* < 0.05, Figure 2C) compared to the non-denervated rats of IR injury; however, the difference in NGAL between the two groups was not statistically significant (106.1 ± 24.2 vs. 61.98 ± 14.53, *P* > 0.05, Figure 2D). Histopathological examination of kidney tissue by H&E staining revealed severe lesion in rats both from IR group and IR + SCGx group. As shown in Figure 2E, the IR group showed severe tubular lysis, loss of brush border, inflammatory cell infiltration, and sloughed debris in the tubular lumen space, as compared with the sham group. SCGx to ischemic acute kidney injury further promoted the development of all these lesions (Figures 2E,F). Results indicate that sympathetic denervation by SCGx aggravates AKI induced by IR injury.

### Sympathetic Denervation Enhanced Renal Ischemic Reperfusion-Induce Inflammation Response and Expression of Pro-apoptosis Protein After Ischemic Reperfusion Injury

**FIGURE 3:**
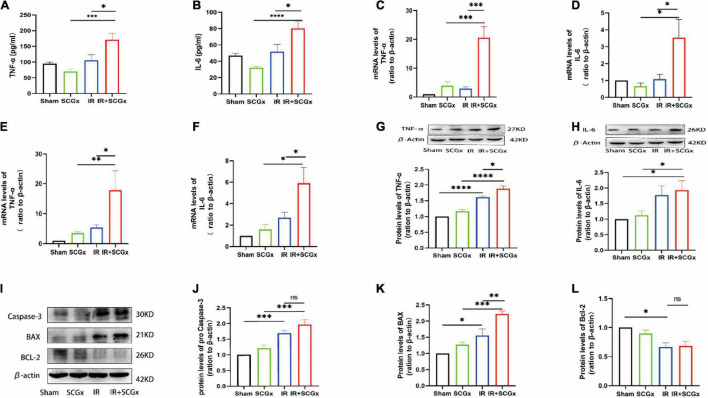
Enhanced inflammation response and expression of pro-apoptosis protein upon sympathetic denervation. **(A,B)** The TNF-α and IL-6 are concentrated in plasma by ELISA. **(C,D)** Real-Time PCR for TNF-α and IL-6 in the hypothalamus. β-actin used as internal control. **(E,F)** Real-Time PCR for TNF-α and IL-6 in the kidney. β-actin used as internal control. **(G,H)** The protein levels of TNF-α and IL-6 in the kidney. β-actin served as an internal control. **(I)** Representative images of protein levels of Bax, Bcl-2 and pro Caspase-3. **(J–L)** Expression levels of Bax, Bcl-2 and Caspase-3 in the kidney were normalized to β-actin levels within the same sample. *P < 0.05; **P < 0.01; ***P < 0.001; ****P < 0.0001, each group contains at least 4 rats.

Inflammation and apoptosis are key factors in the development of renal IR injury, we, therefore, decided to test whether they are affected by sympathetic denervation. According to Enzyme-Linked Immunosorbent Assay (ELISA) results, the inflammatory cytokine levels of TNF-α and IL-6 were significantly higher in the IR + SCGx group than the IR group (TNF-α 171.2 ± 20.74 vs. 105.5 ± 18.17, *P* < 0.05, Figure 3A; IL-6 80.2 ± 8.689 vs. 51.74 ± 8.969, *P* < 0.05, Figure 3B). Likewise, the mRNA levels of pro-inflammation cytokines were found to be clearly increased in IR rats with sympathetic denervation in the hypothalamus, compared with the non-denervated IR rats (TNF-α 20.57 ± 3.969 vs. 2.887 ± 0.6566, *P* < 0.0001, Figure 3C; IL-6 3.538 ± 1.076 vs. 1.082 ± 0.2916, *P*< 0.05, Figure 3D). In addition, the mRNA and protein levels of TNF-α and IL-6 in the kidney were also determined and remarkedly increased in IR rats with sympathetic denervation (mRNA TNF-α 17.84 ± 6.476 vs. 5.409 ± 0.9479, *P* < 0.05, Figure 3E; IL-6 5.918 ± 1.455 vs. 2.697 ± 0.5214, *P* < 0.05, Figure 3F; protein TNF-α 1.878 ± 0.08540 vs. 1.608 ± 0.03856, *P* < 0.05, Figure 3G; IL-6 1.932 ± 0.3039 vs. 1.767 ± 0.3091, *P* > 0.05, Figure 3H). In the subsequent experiment, we examined the expression levels of apoptosis-related proteins, such as Bax, Bcl-2 and Caspase-3, and found that sympathetic denervation also increased levels of Bax in the kidney after IR injury, in spite of no statistical significance in caspase-3, whereas the down-regulated expression of Bcl-2 induced by IR was not affected, compared with the IR group (Bax 2.221 ± 0.09131 vs. 1.562 ± 0.1927, *P* < 0.01; Caspase-3 1.962 ± 0.1487 vs. 1.689 ± 0.08954, *P* > 0.05; Bcl-2 0.6831 ± 0.08026 vs. 0.6666 ± 0.07026, *P* > 0.05, Figures 3I–L).

### Sympathetic Denervation Enhanced the Expression of P2X3R and P2X7R in the Spinal Cord After Renal Ischemic Reperfusion Injury

**FIGURE 4:**
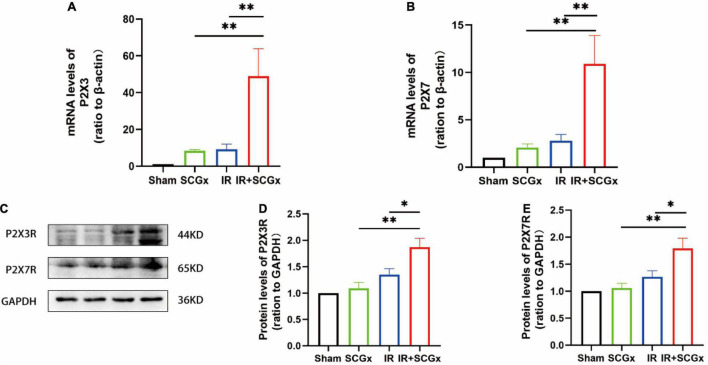
The activated purinergic P2X receptor in the spinal cord upon sympathetic denervation. **(A,B)** Real-Time PCR for P2X3R and P2X7R in the spinal cord. β-actin used as internal control. **(C)** Representative images of protein levels of P2X3R and P2X7R. **(D,E)** The expression levels of P2X3R and P2X7R in the spinal cord were normalized to GAPDH within the same sample. *P< 0.05; ^**^P < 0.01, each group contains at least 4 rats.

In the superior cervical sympathetic ganglion, P2X3 and P2X7 receptors mediated the sympathoexcitatory reflex induced by myocardial ischemic injury (39–41). T_8–12_ spinal cord segments are primarily involved in the sympathetic regulation of renal function (42). To demonstrate whether these purinergic receptors are also involved in the pathophysiology of the adverse effect of sympathetic denervation on renal IR injury, we examined the expression of P2X3R and P2X7R at both mRNA and protein levels. The expression of P2X3R and P2X7R were significantly increased in denervated rats of the IR-induced AKI, compared with non-denervated IR rats (P2X3R mRNA 49.03 ± 14.89 vs. 9.229 ± 2.771, *P* < 0.01; protein 1.87 ± 0.1658 vs. 1.353 ± 0.1131, *P* < 0.05; P2X7R mRNA 10.9 ± 2.994 vs. 1.353 ± 0.1131, *P* <0.01; protein 1.788 ± 0.1931 vs. 1.282 ± 0.08897, *P* < 0.05, Figure 4). However, the protein levels of P2X3R and P2X7R in kidney showed no significantly difference in IR+SCGx group and IR group (Supplementary Figure S1).

## Discussion

The major results of the present study are as follows: (1) Increased sympathetic activity in the kidney and hypothalamus induced by renal IR injury were suppressed following the induction of sympathetic denervation by SCGx in IR rats. (2) The functional and structural damage in IR rats was aggravated by SCGx. (3) Excessive expression of pro-inflammation cytokines and apoptotic protein were observed in denervated rats of AKI induced by IR injury. (4) Those effects brought by the SCGx may have resulted from the activated P2X3R and P2X7R in the spinal cord.

The renal nerves consist of both efferent and afferent nerve fibers. Tissue damage associated with ischemia results in the activation of primary afferent nerve fiber terminals and the transference of signals to the central nervous system, followed by the efferent nerve fiber activated and responsible for modulating renal hemodynamics and renal function. The efferent renal innervation contains postganglionic sympathetic fibers that typically exert their effects through release of NE onto the postsynaptic adrenoceptor (43). Moreover, the majority of experimental research support the evidence that the SNS activity is elevated in response to acute IR (11, 44, 45). The activation of renal sympathetic nerves occurs in acute kidney injury and has been identified as a key pathophysiologic factor contributing to disease progression (46, 47). Here, we carried out sympathectomy by bilateral ganglionectomy of SCG that could suppress the augmented sympathetic activity after renal IR injury and regulate the renal dysfunction and tissue injury during the early post-ischemic phase of renal IR injury.

Most previous studies have supported that the renal venous plasma NE concentration and TH expression is elevated in response to the reperfusion of the ischemic kidney (44, 45), as well as increased sympathetic activity in the cerebral sympathetic regulatory regions (48). Consistent with the above findings, our data also confirmed the presence of sympathetic overactivation after IR injury. In the present study, we obtained evidence that the expression of TH, the rate-limiting enzyme in the biosynthesis of catecholamine, was significantly elevated in the kidney and hypothalamus after IR injury, although the plasma NE concentration by ELISA was not changed. We also found that increases in TH were suppressed by SCGx. Interestingly, NE content notably decreased followed by sympathetic denervation in postischemic rats.

It has long been considered that, inhibition of the SNS is the target of a wide variety of therapeutic interventions in a number of clinical conditions. Current therapeutic interventions [such as renal denervation (49), ganglionic blockade (14) and centrally acting sympatholytic drugs (50)] have been shown to attenuate renal dysfunction and histological damage in experimental IR rats, mice and rabbits. Salman et al. reported that renal denervation before ischemia attenuated the deteriorated renal hemodynamic and excretory functions during the early post-ischemic phase of renal IR injury (49). Notably, an earlier study reported that GABA content increased after chronic SCGx in the mediobasal and anterior hypothalamic (51), while GABA was demonstrated to have protective effect on IR-induced renal injury in rats (52). In contrast, with regard to the effect of sympathetic denervation by SCGx on the progression of IR-induced AKI injury, our study showed aggravated renal morphological and functional changes in denervated rats with renal IR injury, manifested by the elevated biochemical markers such as BUN, KIM-1 and NGAL, as well as severe histopathological alteration.

Evidence also suggests, that there is a neuroimmune bidirectional network between the immune and SNS (53). Inflammation response resulting from acute kidney injury/acute stress is associated with suppressed parasympathetic nervous system activity and prevalence of the activation of the SNS. Furthermore, systemic and tissue release of NE, the primary neurotransmitter of the SNS coupled with the specific receptor expression in immune cells, can exert marked effects on the inflammation/immune response, such as modulation of cytokine production (54). A previous study showed augmented TNF-α secretion by peritoneal macrophages observed in sympathectomized mice (55). Similar exacerbation of TNF-α production after liver injury in chemically sympathectomized mice was reported by other investigators (56). Electrical stimulation of sympathetic nerves is also known to directly inhibit LPS-induced TNF-α secretion (57). However, Zhou et al. showed that NE upregulated TNF-α production *in vitro* experiments (58). Taken together, conclusions from the above studies seem to be contradictory, probably because of the differential effects mediated by the specific adrenergic receptor subtype. It has been suggested that the anti-inflammation effects of NE appear to be mediated via β_2_-adrenergic receptors, in which NE inhibits proinflammatory cytokines and stimulates the production of anti-inflammatory cytokines through β_2_-adrenergic receptor-cAMP-protein kinase A pathway (59). Whereas, α_2_-adrenoceptro activation was demonstrated to have pro-inflammation effects (60). On the other hand, electrical stimulation of the vagus nerve inhibited synthesis of TNF-α in the liver, spleen and heart, and attenuated serum concentration of TNF during endotoxemia by means of the so-called “cholinergic anti-inflammation pathway” (61). The disparate actions of the SNS may depend on the degree and duration of its activation with short activation periods having anti-inflammatory effects and sustained activation showing pro-inflammatory effects (14). Therefore, the role of the sympathetic system involved in regulating the immune system needs to be further studied.

To investigate the specific mechanism, we examined the expression levels of associated pro-inflammatory factors. Evidence from research has indicated that sympathetic overactivity aggravates IR-induced renal damage via pro-inflammation mechanisms (10, 11). And several lines of other evidence suggest that peripheral production of pro-inflammation cytokines can signal the brain and alter neural signaling in the hypothalamus (62–64). In the current study, AKI did result in significant increase in levels of TNF-α and IL-6 in the blood, kidney and hypothalamus at 24 h after ischemia. Furthermore, our data obtained with sympathetic denervation by SCGx showed enhanced inflammation response after renal IR injury. Consistent with our findings, a pervious study by Grigoryev et al. demonstrated that IR-induced AKI leads to vigorous inflammation response in blood and lungs (65). Another study reported that ischemic AKI markedly increased brain inflammation evidenced by increased pro-inflammation cytokines, glial activation, and disrupted blood-brain barrier (66). The central nervous system receives sensory input from the immune system and responds to increased levels of circulating TNF-α, and activation of the hypothalamus-pituitary response to renal IR injury. Over and above, Martín et al. concluded that peripheral sympathetic nerve terminals originating in the SCG may modulate acute stress responses (67), which may help to explain the effect of SCGx on renal IR injury. Apoptosis, likewise, is a marker of tissue injury in AKI. In the present study, we also investigated apoptosis activity and found the expression of the pro-apoptosis protein to be increased, such as Bax, which provide greater insights of the deleterious effect of SCGx against renal IR injury.

Renal epithelial cells contain abundant ATP, and during acute renal ischemia, the visceral spinal afferent neurons including nociceptors can be activated by the ATP released from the damage kidney (68). The biological actions of extracellular ATP are mediated by purinergic receptors, including P2XRs and P2YRs. The P2X receptors, consist of seven subtypes (P2X1-7), which are involved in a variety of biological responses, but mainly associated with inflammation, tissue damage and cell proliferation (69). With respect to the P2X3 receptor, it is highly and selectively expressed in nociceptive sensory neurons, and could be activated by chemical mediators released from damaged tissue such as CGPR, thus, plays a crucial role in the processing of sensory inputs in the spinal cord (70). The association of the P2X7R with inflammation is long-standing. The P2X7R promotes the release of IL-6 and TNF from mouse microglia (71), which are implicated in tubular fibrosis and apoptosis in response to ureteral obstruction in mice (72). A recent report suggests that P2X7R activation accelerates the development of AKI by potentiating renal tubular cell death and the inflammation response (73). In our data, sympathetic denervation by SCGx markedly upregulated the expression of P2X3R and P2X7R at both mRNA and protein in T_8–12_ spinal cord segments after renal IR injury. This is consistent with other studies that demonstrated that P2X3R and P2X7R in the superior cervical ganglion are involved with the increased sympathoexcitatory reflex induced by myocardial ischemia (39–41). Thus, our results indicated that sympathetic denervation by SCGx regulated sympathoexcitatory reflex in renal ischemic reperfusion probably through purinergic signaling.

## Conclusion

In summary, our study clearly indicates that surgical removal of SCG resulted in decreased norepinephrine overflow and reduced sympathetic hyperactivation in the kidney and hypothalamus after renal IR injury. Although such sympathetic denervation process aggravates IR-induced AKI in rats, the underlying mechanism may involve the modulation of inflammation and the activated purinergic signaling in the spinal cord segments that dominate the kidney.

## Data Availability Statement

The original contributions presented in the study are included in the article/Supplementary Material, further inquiries can be directed to the corresponding author/s.

## Ethics Statement

The animal study was reviewed and approved by the Institutional Ethical Committee of Tongji Hospital, Tongji Medical College, Huazhong University of Science and Technology (Wuhan, China).

## Author Contributions

All authors listed have made a substantial, direct, and intellectual contribution to the work, and approved it for publication.

## Conflict of Interest

The authors declare that the research was conducted in the absence of any commercial or financial relationships that could be construed as a potential conflict of interest.

## Publisher’s Note

All claims expressed in this article are solely those of the authors and do not necessarily represent those of their affiliated organizations, or those of the publisher, the editors and the reviewers. Any product that may be evaluated in this article, or claim that may be made by its manufacturer, is not guaranteed or endorsed by the publisher.
