# Supplementary material for: The Role of the Superior Cervical Sympathetic Ganglion in Ischemia Reperfusion-Induced Acute Kidney Injury in Rats
Source: Front Med (Lausanne). 2022 Apr 21;9:792000. doi: 10.3389/fmed.2022.792000 (PMC9069004; doi:10.3389/fmed.2022.792000)
Supplement: Supplementary file 1 [file Data_Sheet_1.pdf]

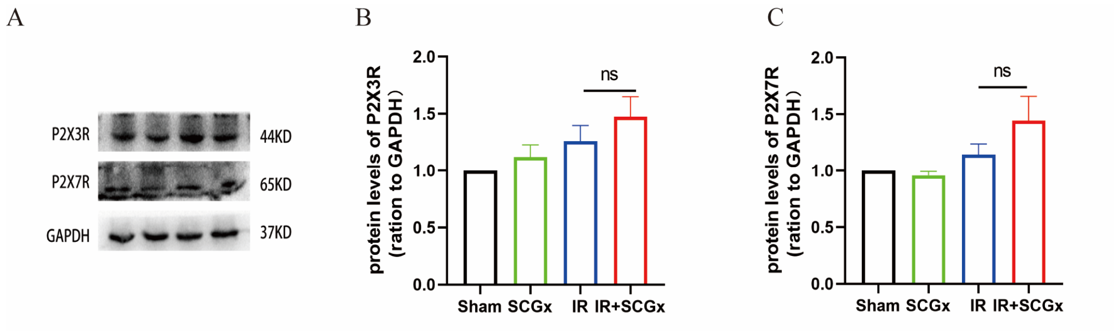


**Figure S1. The protein levels of the purinergic P2X receptor in the kidney.** (A) Representative images of protein levels of P2X3R and P2X7R. (B, C) The expression levels of P2X3R and P2X7R in the kidney were normalized to GAPDH within the same sample. GAPGH served as an internal control. "ns", $P > 0.05$, each group contains at least 4 rats.
